# Supplementary material for: Drug-eluting versus bare-metal stents for first myocardial infarction in patients with atrial fibrillation: A nationwide population-based cohort study
Source: PLoS One. 2020 Jan 10;15(1):e0227571. doi: 10.1371/journal.pone.0227571 (PMC6953848; doi:10.1371/journal.pone.0227571)
Supplement: S1 Table — (DOC) [file pone.0227571.s001.doc]

**Supporting information**

**S1 Table. ICD-9-CM code**

ICD-9-CM, International Classification of Diseases–Ninth Revision–Clinical Modification.

| Variable | Code |
| --- | --- |
| Acute myocardial infarction | 410.xx |
| Atrial fibrillation | 427.3x |
| Hypertension | 401.xx–405.xx |
| Diabetes mellitus | 250.xx |
| Dyslipidemia | 272.xx |
| Coronary artery disease | 410.xx–414.xx |
| Heart failure | 428.xx |
| Chronic kidney disease | 580.xx–589.xx–403.xx–404.xx–016.0x–095.4x–236.9x–250.4x–274.1x–442.1x–447.3x–440.1x–572.4x–642.1x–646.2x–753.1x–283.11–403.01–404.02–446.21 |
| Dialysis | 585.xx (Catastrophic illness card) |
| Gout | 274.xx |
| Chronic obstructive pulmonary disease | 491.xx–492.xx–496.xx |
| Peripheral arterial disease | 440.0–440.2x–440.3x–440.4–440.9–443.9–444.2–444.22–444.8–444.81–445.0–445.02–250.7x–707.1x |
| Malignancy | 140.xx–208.xx (Catastrophic illness card) |
| Old myocardial infarction | 410.xx–412.xx |
| Stroke | 430.xx–437.xx |
| Acute kidney injury | 584.xx |
| Gastrointestinal bleeding | 530.21–530.7–530.82–531.xx–534.xx–535.xx–537.83–537.84–578.xx |
| Major bleeding | 430.xx–432.xx–578.xx–719.1x–423.0–599.7–626.2–626.6–626.8–627.0–627.1–786.3–784.7–459.0 |
| Ischemic stroke | 433.xx–437.xx |
| Hemorrhagic stroke | 430.xx–432.xx |
| Unspecified stroke | 436.xx–437.xx |
